# Effects of low-dose ibuprofen supplementation and resistance training on bone and muscle in postmenopausal women: A randomized controlled trial

**DOI:** 10.1016/j.bonr.2016.04.004

**Published:** 2016-04-28

**Authors:** Whitney R.D. Duff, Saija A. Kontulainen, Darren G. Candow, Julianne J. Gordon, Riley S. Mason, Regina Taylor-Gjevre, Bindu Nair, Michael Szafron, Adam D.G. Baxter-Jones, Gordon A. Zello, Philip D. Chilibeck

**Affiliations:** a College of Kinesiology, Physical Activity Complex, 87 Campus Drive, University of Saskatchewan, Saskatoon, SK S7N 5B2, Canada; b College of Medicine, Health Sciences Building Box 19, 107 Wiggins Road, University of Saskatchewan, Saskatoon, SK, S7N 5E5, Canada; c School of Public Health, Health Sciences Building, 104 Clinic Place, University of Saskatchewan, Saskatoon, SK, S7N 2Z4, Canada; d College of Pharmacy and Nutrition, Health Sciences Building, 104 Clinic Place, University of Saskatchewan, Saskatoon, SK, S7N 2Z4, Canada; e Faculty of Kinesiology and Health Studies, Centre for Kinesiology, Health, and Sport, 3737 Wascana Parkway, University of Regina, Regina, SK, S4S 0A2, Canada

**Keywords:** Aging, Osteoporosis, Sarcopenia, Ibuprofen

## Abstract

**Purpose:**

To compare the effects of nine months of exercise training and ibuprofen supplementation (given immeditately after exercise sessions) on bone and muscle in postmenopausal women.

**Methods:**

In a double-blind randomized trial, participants (females: n = 90, mean age 64.8, SD 4.3 years) were assigned (computer generated, double blind) to receive supervised resistance training or stretching 3 days/week, and ibuprofen (400 mg, post-exercise) or placebo (i.e. 4 groups) for 9 months. In this proof-of-concept study the sample size was halved from required 200 identified via 90% power calculation. Baseline and post-intervention testing included: Dual energy x-ray absorptiometry (DXA) for lumbar spine, femoral neck, and total body areal bone mineral density (aBMD); geometry of proximal femur; total body lean tissue and fat mass; predicted 1-repetition maximum muscle strength testing (1RM; biceps curl, hack squat).

**Results:**

Exercise training or ibuprofen supplementation had no effects on aBMD of the lumbar spine, femoral neck, and total body. There was a significant exercise × supplement × time interaction for aBMD of Ward's region of the femoral neck (p = 0.015) with post hoc comparison showing a 6% decrease for stretching with placebo vs. a 3% increase for stretching with ibuprofen (p = 0.017). Resistance training increased biceps curl and hack squat strength vs. stretching (22% vs. 4% and 114% vs. 12%, respectively) (p < 0.01) and decreased percent body fat compared to stretching (2% vs. 0%) (p < 0.05).

**Conclusions:**

Ibuprofen supplementation provided some benefits to bone when taken independent of exercise training in postmenopausal women. This study provides evidence towards a novel, easily accessible stimulus for enhancing bone health [i.e. ibuprofen].

## Highlights

• Ibuprofen independently (i.e. without exercise) may have some benefits on bone.
• Ibuprofen was not effective if given immediately after resistance training.
• Ibuprofen may be a novel therapy to prevent bone loss associated with inflammation.

## 1 Introduction

Inflammation is considered a main pathophysiological contributor to sarcopenia (i.e., loss of muscle mass and muscle function) (J Am Med Dir Assoc, 2011) and osteoporosis (i.e., loss of bone mass and bone strength) (Med Sci Sports Exerc, 2009). Sarcopenia and osteoporosis are associated with frailty and functional impairment, resulting in the decreased capacity to perform daily living activities, ultimately impacting on quality of life and increasing mortality (International Working Group on Sarcopenia, 2011, De Martinis et al., 2006). Resistance training is an effective intervention for increasing muscle and bone mass; however, aging individuals experience an attenuated response to resistance training which contributes to aging anabolic resistance and sarcopenia (Breen and Philips, 2011). Furthermore, the increase in areal bone mineral density (aBMD) at clinically relevant sites such as the lumbar spine and femoral neck with resistance training are modest (~ 1–2%) but nonetheless beneficial (Gomez-Cabello et al., 2012, Kohrt et al., 2013). Therefore, a longer-term (i.e. > 6 months) resistance training program combined with additional interventions may be required to produce significant muscle and/or bone benefits in aging adults.

Ibuprofen is a non-steroidal anti-inflammatory drug (NSAID) known for its anti-inflammatory properties via reductions of prostanoids derived from reactions catalyzed by the cyclooxygenase (COX-1 and COX-2) enzymes (Rainsford, 2009). Ibuprofen supplementation attenuates the loss of muscle mass in animal models of aging (McCarthy et al., 2004, Rieu et al., 2009). Epidemiological studies in humans demonstrate associations between regular NSAID use and moderate (~ 2–6%) increases in aBMD at clinically relevant sites (Bauer et al., 1996, Carbone et al., 2003, Morton et al., 1998, Richards et al., 2006). A systematic review suggests that the beneficial effects of NSAIDs on bone health may be due to the attenuation of inflammatory cytokines inhibiting bone resorption (Konstantinidis et al., 2013). Collectively, ibuprofen may reduce inflammation associated muscle and bone loss in aging humans.

Evidence of the combined effects of exercise and ibuprofen supplementation is limited. When ibuprofen is administered following exercise training, the inhibition of prostaglandins (PGE_2_) may have beneficial effects on muscle and bone health via prevention of protein catabolism, resulting in muscle accretion and protein retention in older adults (Trappe et al., 2011); and inhibition of the altered inflammatory response of COX-2 and prostaglandin E_2_ (PGE_2_) after the mechanically induced bone formation has already occurred (Kohrt et al., 2013, Kohrt et al., 2010). A high dose of ibuprofen (1200 mg daily) is effective for increasing muscle size and strength in older adults over a short resistance training period (12 weeks) (Trappe et al., 2011). A lower dose of ibuprofen (400 mg) administered only after resistance training (3–5 days/week) over a shorter training period was not effective in increasing lean tissue mass or muscle strength in younger individuals or older women (Candow et al., 2013, Krentz et al., 2008); however, it was effective for increasing the aBMD of clinically relevant sites in premenopausal women during a longer training period (9 months) (Kohrt et al., 2010). While ibuprofen given after resistance training appears beneficial for bone in *premenopausal* women, this was not evident in a recent study of *postmenopausal* women over a similar training period (Jankowski et al., 2015). Research over longer training periods in older women remains scarce, with the only notable study lacking an exercise control group, thus not allowing for the determination of the interaction between resistance training and ibuprofen (Jankowski et al., 2015).

Additional anti-inflammatory interventions combined with resistance training are needed to determine if there are clinically relevant improvements in aging muscle and bone. The purpose of our study was to investigate the effects of a long term (9 months) intervention of combined ibuprofen (400 mg) and exercise training on muscle and bone in postmenopausal women (60 y or greater). We hypothesized a combined effect of progressive resistance training and ibuprofen supplementation leading to improved lean tissue mass and bone properties.

## 2 Participants and methods

### 2.1 Study design

A proof-of-concept, double-blind (for ibuprofen), factorial randomized control trial design was employed to compare the independent and combined effects of ibuprofen supplementation and exercise training. Participants were randomized 1:1:1:1 to one of four groups after being cleared for inclusion. Randomization was completed using a computer-generated allocation schedule with a block size of four by one of the investigators who was not involved in the measurement of outcome variables or the analysis. The four unique groups were: 1) resistance training combined with ibuprofen supplementation (ExIbu); 2) resistance training combined with placebo supplementation (Ex); 3) flexibility training (exercise placebo) combined with ibuprofen supplementation (Ibu); and 4) flexibility training combined with placebo supplementation (Control). Ibuprofen dosage was 400 mg after exercise training only (maximum 3 times per week) for 9 months. This dose and length of supplementation was chosen because it is safe and effective for increasing aBMD (hip region) when taken after resistance training in premenopausal women (Kohrt et al., 2010). Further, this dosage was well-tolerated in a 9 week pilot study of postmenopausal women completed by our research group (Candow et al., 2013). Ibuprofen and placebo (methylcellulose) were administered in a double-blind fashion in the form of capsules that were identical in taste, color, and appearance. The supplement was pre-packaged into containers that were sequentially numbered according to the randomization schedule, of which the allocation sequence was concealed from the research assistants enrolling and assessing the participants. After completion of the baseline testing, participants were provided containers with the supplement (i.e. ibuprofen or placebo), calcium and vitamin D (600 mg/d and 400 IU/d, respectively), and an exercise/supplement tracking log. Participants in the stretching group took their supplement at home while participants in the resistance training group took their supplement as provided directly by a research assistant after each resistance training session as post-exercise ibuprofen has a beneficial effect on aBMD (Kohrt et al., 2010). Calcium and Vitamin D was taken daily at home by all participants. Although participants could not be blinded to the exercise assignment, they were blinded to the hypothesis that the resistance training would be superior to flexibility training. All the researchers involved in the outcome assessment and analysis were blinded to the group assignment. The personnel supervising the training program were blinded to the supplement (i.e. ibuprofen or placebo). Statistical analysis was blinded through the coding of the groups. The study was approved by the Biomedical Research Ethics Board of the University of Saskatchewan. Reporting of this study adhered to the Consolidated Standards of Reporting Trials (CONSORT) guidelines for randomized clinical trials. This trial was registered with clinicaltrials.gov (NCT01886196).

### 2.2 Participants

Postmenopausal women 60 years or older were recruited via advertisements in local newspapers and posters from January 2013 to September 2013. All 164 potential participants that responded to the advertisements were assessed for study eligibility by modification of the Mediterranean Osteoporosis Study Questionnaire (MEDOS) (Dequeker et al., 1991). Only subsections of MEDOS important for this study were addressed (i.e. diseases/medications applicable to bone/ibuprofen). Grounds for exclusion included usage of medication or presentation of disease that is known to affect bone mineral metabolism. Thus, participants with Crohn's Disease or Cushing Disease, currently taking systemic corticosteroids, or having taken bisphosphonates, hormone replacement therapy, selective estrogen receptor modulators, parathyroid hormone, or calcitonin within the past 12 months were excluded. Participants were also excluded if they were currently taking medication or had presentation of disease that is known to interfere with ibuprofen. Thus, participants with severe osteoarthritis or severe heartburn, ulcers, or gastritis requiring acid reducers (e.g. H2 blockers or proton pump inhibitors), currently taking NSAID (e.g. prophylactic acetylsalicylic acid) or blood thinners due to past episodes of deep vein thrombosis or pulmonary embolism were excluded. After clearing initial exclusion criteria, participants were further evaluated for 10-year risk of fracture based on age and femoral neck aBMD t-score (i.e. Canadian Association of Radiologists and Osteoporosis Canada [CAROC]) and excluded if they were classified as “high” risk for fracture (Can Med Assoc J, 2010). The CAROC method classifies individuals as being at “low”, “moderate”, or “high risk” for fracture, where fragility fracture or systemic corticosteroid use (i.e. a prednisone equivalent dosage of ≥ 7.5 mg/day for at least three cumulative months during the preceding year) moves the individual up one risk category (Can Med Assoc J, 2010). Participants were instructed not to ingest any type of NSAID for the duration of the study. Finally, participants were excluded if they were active smokers or were currently taking part in a moderate to vigorous resistance-training program more than once per week.

**Fig. 1:**
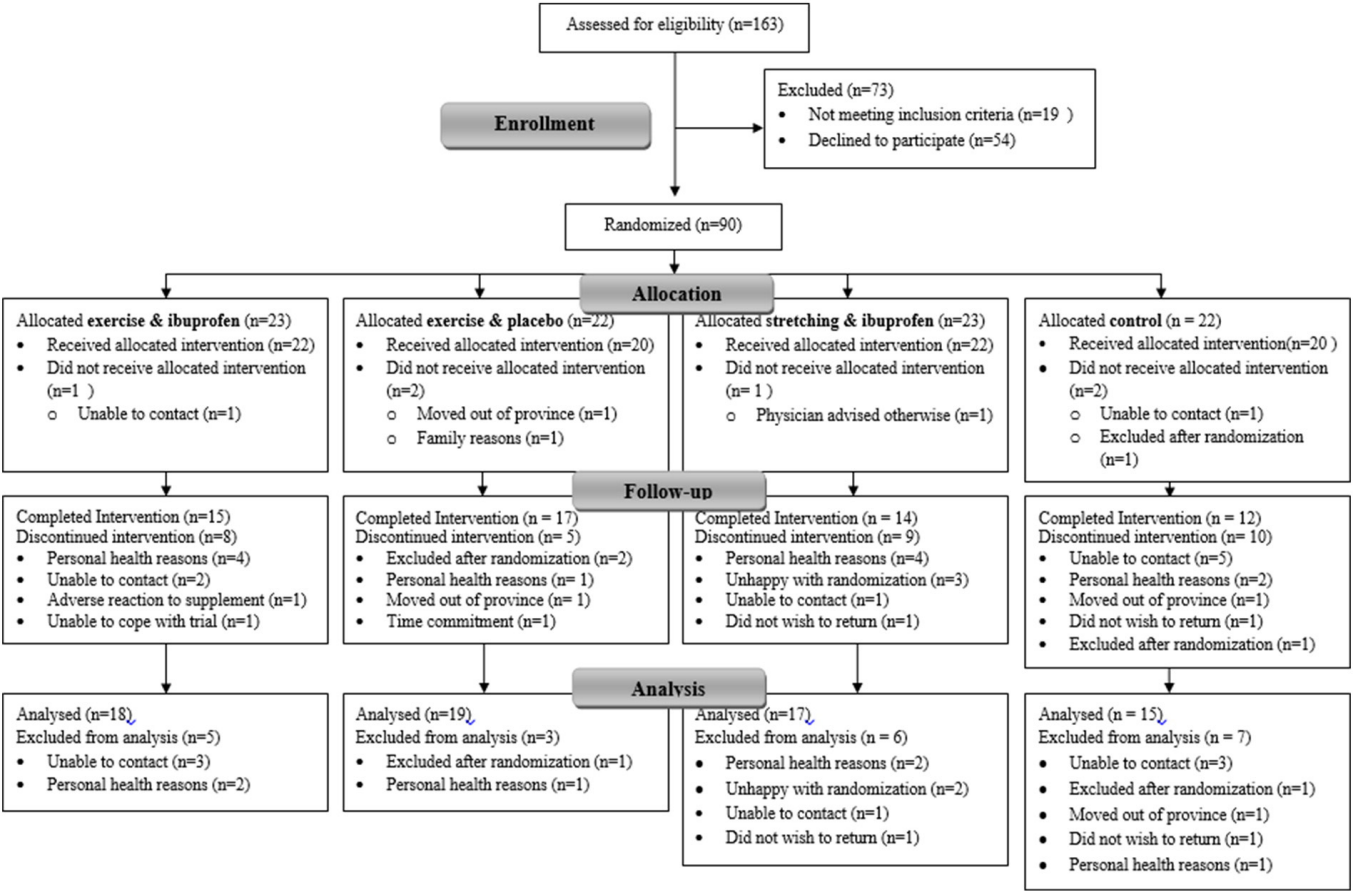
CONSORT Flow diagram. Participant flow throughout duration of study.

After applying the exclusion criteria, 144 women were eligible and 90 decided to participate in the study (see Fig. 1 for flow diagram of participants). To detect clinically relevant differences in primary variables with 90% power (2 sided, p ≤ 0.05), and accommodate 20% attrition, each group would require approximately 50 per-protocol participants (Jankowski et al., 2015). Due to the proof-of-concept nature of this study the target sample size identified via power calculations was halved. Further justification for the sample size of this proof-of-concept study was based on a previous intervention of young individuals (n = 54) with beneficial aBMD response to NSAID supplementation after exercise (Kohrt et al., 2010) and increased because older individuals have greater variability in their physiological measurements (Candow and Chilibeck, 2005). Participants were randomized into 4 groups, as described in the study design (Fig. 1).

Participants signed informed consents and completed the Physical Activity Readiness Questionnaire (PAR-Q) (Thomas et al., 1992) prior to baseline testing to ensure there was no contra-indication to exercise participation. Those with a positive response to the PAR-Q and those over the age of 69 years were required to have their physician complete the Physical Activity Readiness Medical Examination (PARmedX) prior to participation. All participants completed the intervention by July 2014.

### 2.3 Interventions

Ibuprofen and placebo obtained from the Saskatoon Medical Arts Pharmacy (Saskatoon, SK) were administered orally in identical capsules at a dose of 400 mg immediately following exercise training (resistance and flexibility training) 3 days per week maximum. The contents of the ibuprofen (96% ibuprofen) were verified through independent laboratory testing (Eagle Analytical Services, Houston, TX). The placebo capsule contained methylcellulose that was indistinguishable in appearance from the ibuprofen. All participants received a supplement of 600 mg of calcium and 10 μg (400 I.U.) of vitamin D per day in the form of a pill or chew (Jamieson Laboratories, Toronto, ON) to assist in meeting the Osteoporosis Society of Canada recommendations of 1200 mg per day for calcium and 20 μg (800 I.U.) per day for vitamin D (Can Med Assoc J, 2010).

The exercise intervention consisted of resistance training performed 3 days per week on non-consecutive days. Prior to their first exercise session, participants attended an orientation session in our research gymnasium to be familiarized with the exercises and machines. Orientation and all resistance training exercise sessions were completed under the supervision of a Canadian Society for Exercise Physiology-Certified Exercise Physiologist research assistant (www.csep.ca). The resistance training exercise intervention required 2 sets of 8–12 repetitions of 12 exercises designed to train all major muscle groups. Exercises were performed on Lever machines (Pulse Fitness Systems; Winnipeg, Manitoba, Canada) or with free weights. Exercises performed on machines included: hack squat, hip flexion, extension, adduction, and abduction, and dorsiflexion. Exercises performed with dumbbells included: biceps curl, forearm curl, supinated wrist curl, pronated wrist curl, front and side step ups, single leg lunge, and plantar flexion. In addition, participants performed a medicine ball toss and catch against a wall. Participants were encouraged to work to muscle fatigue and monitored to ensure that resistance was increased once two full sets of 12 repetitions could be performed with good form. Participants were required to sign in for every exercise session and provided with resistance training logs to track sets, loads, and repetitions (i.e. overall volume) and supplement tracking logs to track dosages of supplement and calcium and vitamin D consumption.

The exercise placebo consisted of a home-based flexibility program performed 3 days per week on non-consecutive days. Flexibility participants completed an orientation at our research gymnasium and were provided with a print version of the home-based program. The exercise placebo intervention required 2 sets held for 20–30 s of full body flexibility (i.e. stretching) exercises. The exercise placebo groups were contacted monthly to assess compliance to the program and monitor adverse events. Flexibility participants were advised not to perform any resistance training exercise for the duration of the intervention. Compliance to the exercise intervention was assessed by attendance at the supervised exercise sessions (Ex and ExIbu) and tracking logs for the home-based flexibility program (Ibu and Control). Adverse events for all groups during the intervention were recorded on an adverse event form. Compliance to the supplement and calcium and vitamin D for all groups was assessed via tracking logs and amount of left-over supplement. In addition, prior to being ‘unblinded’, to assess the effectiveness of the blinding, participants were questioned regarding what supplement they thought they were on. The duration of the intervention was 9 months.

### 2.4 Outcomes

All outcome measurements were completed at baseline and 9 months (Kohrt et al., 2010). Primary outcomes were the aBMD of the proximal femur and lumbar spine. Secondary outcomes were: cross-sectional area (CSA), subperiosteal width (SPW), and section modulus (Z) of the narrow part of the femoral neck, the intertrochanter region, and the shaft of the proximal part of the femur; total body aBMD, lean tissue and fat mass; biceps curl and hack squat muscular strength; and backwards tandem walking balance performance. Tertiary outcomes were adverse events. Dual energy x-ray absorptiometry (DXA) scans were performed prior to any physical testing to determine study eligibility. Following the DXA scan, participants completed balance testing, and then performed a standardized warm-up prior to any strength tests. The tests were performed in this particular order to prevent balance testing being compromised due to muscle fatigue resulting from strength testing.

#### 2.4.1 Dual energy x-ray absorptiometry (DXA)

Areal bone mineral density and body composition were assessed via DXA in array mode (QDR Discovery Wi; Hologic, Inc., Bedford, MD, USA) using QDR software for Windows XP (QDR Discovery). Sites measured included lumbar spine (L_1_–L_4_ vertebrae), proximal femur (total hip, femoral neck, trochanter, intertrochanter, and Ward's region), and total body. The coefficients of variation for lumbar spine, proximal femur and total body aBMD in our laboratory are 0.7%, 1.0%, and 0.5%, respectively (Chilibeck et al., 2013). Hip Structural Analysis (HSA) was used for assessment of CSA, SPW, and Z of the narrow neck (NN), intertrochanter (IT) and femoral shaft (FS) regions (Beck et al., 1990). All DXA analyses were performed by a blinded study radiologist. Bone CSA is representative of bone mineral mass within a cross-section in terms of the cortical equivalent surface area and indicative of bone compressive strength. SPW is measured as the outer diameter of the bone computed as the blur-corrected width of the mass profile. Section Modulus (Z) provides an estimate of bone bending strength (Beck et al., 1990). The coefficients of variation for NN, IT, and FS regions, respectively, for our laboratory are as follows: CSA (2.6%, 2.2%, and 1.8%); SPW (5.3%, 1.8%, and 1.2%); and Z (3.5%, 3.4%, and 2.1%) (Chilibeck et al., 2013). Body composition, including fat free mass (i.e. lean mass), fat mass, and percent body fat, was also assessed via DXA scan. The coefficients of variation for lean mass and fat mass are 1.0% and 3.0%, respectively (Chilibeck et al., 2013).

#### 2.4.2 Backward tandem walk

Dynamic balance was assessed, taking into account errors made, via a timed backward tandem (i.e. heel to toe) walk over a raised 6 m long board (Chilibeck et al., 2013). The time to cover the distance and number of errors (i.e. number of times stepping off the board) were averaged over two trials.

#### 2.4.3 Submaximal prediction of 1-repetition maximum

Upper and lower body strength was assessed via the submaximal prediction of 1-Repetition Maximum (1RM). Participants were shown proper form and breathing prior to performing a muscle-specific warm-up. For upper body warm-up, testers selected a load for biceps curl that the participant could easily perform 8 repetitions with proper form. For lower body warm-up, participants performed 8 repetitions on the hack squat machine with no additional load added (i.e. weight of the machine as warm-up weight). Testers then selected an estimated load for upper and lower body test that the participant could perform no more than 10 repetitions with proper form. If 10 repetitions were completed in the first attempt, a one-minute rest period was given, and the process was repeated with a heavier load for a second and/or third attempt. If 10 repetitions were not completed in the first attempt the test was terminated. 1RM was predicted by finding the corresponding percentage of load utilized for number of repetitions completed (e.g. 7 repetitions corresponds to 83%, thus if load utilized was 50 kg then predicted 1RM is 60 kg) (Baechle and Earle, 2000).

### 2.5 Descriptive outcomes

#### 2.5.1 Questionnaires

Participants completed a food frequency questionnaire (FFQ) and leisure time exercise questionnaire (LTEQ) (Godin and Shephard, 1985). The validated FFQ was used to assess the changes from baseline to post-intervention for total energy, calcium and vitamin D intakes based on Canadian dietary reference intakes (Block 98#256318–2; Block Dietary Data Systems, Berkeley, CA, USA). The FFQ, along with directions and images showing sample portion sizes, was sent home with participants to complete and was checked for completeness when returned prior to being sent for computer analysis (Nutrtion Quest, Berkeley, CA, USA; www.nutritionquest.com). The changes in LTEQ from baseline to post-intervention were also determined. The LTEQ assesses non-work related physical activity and exercise training (i.e. cardiorespiratory, resistance, and neuromotor) further categorized as mild, moderate, or strenuous. In older adults the LTEQ has good reliability (test-retest correlation of 0.62–0.74) and has been shown to have a positive association with muscular strength and power (Candow and Chilibeck, 2005, Godin and Shephard, 1985).

#### 2.5.2 Adverse events

Participants were asked to report any adverse events (AE) that occurred throughout the duration of the study. AE forms included a brief description of the event, onset and resolution (unless on-going) dates, rating for seriousness and severity, and relationship to experimental procedure.

### 2.6 Statistical analysis

Data were analyzed on an intent-to-treat basis using IBM SPSS Statistics for Windows (Version 21.0; Armonk, NY: IBM Corp). Baseline characteristics of all variables were compared between groups using Student's *t*-tests. Comparisons of intervention arms were analyzed via a three-factor analysis of variance, with between-group factors for drug (ibuprofen versus placebo) and exercise (resistance training versus flexibility training [exercise placebo]) and one within-subjects factor for time (baseline versus nine months post-intervention). Tetrad contrast hypothesis tests were used for the post-hoc analyses. We report partial eta-squared (ƞ_p_^2^) as estimate of effect size. All descriptive results were expressed as either means and standard deviations or mean absolute changes and 95% confidence intervals. P-values ≤ 0.05 were deemed statistically significant.

## 3 Results

The final analysis included 69 intent-to-treat participants with the remainder lost to follow-up (Fig. 1). In comparison to drop-outs, intent-to-treat participants had lesser fat mass (p = 0.037) and percent body fat (p = 0.004) and were of a taller height (p = 0.016). Baseline descriptives by intervention group are presented in Table 1. Intervention groups were not significantly different for any outcomes at baseline. Reported compliance corresponds to both exercise and supplement as the supplement was only consumed after exercise, and appeared similar between groups (p > 0.05): 1) ExIbu: 89%, 2) Ex: 84%, 3) Ibu: 88%, and 4) Control: 87%. At the end of the study, the percent able to correctly identify the supplement they were blindly receiving was: 1) ExIbu (n = 17): 47%, 2) Ex (n = 19): 63%, 3) Ibu (n = 15): 47%, and 4) Control (n = 14): 79%. The remainder either never began supplementation, guessed incorrectly, or were unable to guess. Compliance to calcium and vitamin D supplementation was similar (p > 0.05) between groups: 1) ExIbu: 83%, 2) Ex: 72%, 3) Ibu: 76%, and 4) Control: 84%. The number of participants analyzed per outcome varied. For hip geometric properties, one (n = 1) scan could not be analyzed for IT variables due to improper positioning. One (n = 1) intent-to-treat participant did not return for a DXA scan, but completed other post-testing outcomes. For the balance tests, some participants (n = 5) could not safely complete the backward tandem walk. For the exercise tests, some participants could not complete the biceps curl 1RM (n = 6) or hack squat 1RM (n = 15) due to injury or refusal. Incomplete questionnaire data for LTEQ (n = 7) and FFQ (n = 8) were due to refusal to complete or return questionnaires.

### 3.1 Bone properties

Group × time interactions were not significant for the aBMD of the lumbar spine, femoral neck, or total body (Table 2), or hip geometric properties (Table 3). For sub-regions of the hip scans, an exercise × supplement × time interaction was significant for aBMD of the Ward's area (p = 0.015; ƞ_p_^2^ = 0.088). When comparing change scores between groups, only the change score for the Ibu group was different from the Control group (p = 0.017). The Control group decreased Ward's region aBMD (Table 2).

### 3.2 Body composition

Group × time interactions were not significant for lean tissue mass; however the exercise × time interaction was significant for fat mass (p = 0.033; ƞ_p_^2^ = 0.069) and percent body fat (p = 0.019; ƞ_p_^2^ = 0.083). Resistance training decreased fat mass and percent body fat compared to stretching (Table 4).

### 3.3 Strength and balance

The exercise × time interaction was significant for hack squat strength and biceps curl strength (p < 0.001). Resistance training increased hack squat (ƞ_p_^2^ = 0.456) and biceps curl (ƞ_p_^2^ = 0.313) strength compared to stretching (Table 4). Group × time interactions were not significant for balance (Table 4).

### 3.4 Diet and activity

The exercise × time interaction was significant for total energy (p = 0.047; ƞ_p_^2^ = 0.068) and fat intake (p = 0.039; ƞ_p_^2^ = 0.073). The stretching group decreased total energy intake via reduced fat intake compared to resistance training (Table 5). The exercise × time × supplement interaction was significant for vitamin D intake (p = 0.024; ƞ_p_^2^ = 0.081), but changes in vitamin D intakes did not differ between the groups in the post-hoc analysis (Table 5). Group × time interactions were not significant for remaining macronutrients, calcium intake, or amount of leisure time exercise performed from baseline to post-intervention (Table 5). The recommended dietary allowances of 0.8 g/kg of protein were met by all groups.

### 3.5 Adverse events

Of the 90 women randomized, two serious adverse events (SAEs) were reported during the intervention; one in a participant assigned to ExIbu and one in a participant assigned to Control. Both SAEs were deemed as “not related” to the intervention; however, both discontinued the intervention. The SAE in the ExIbu participant was a transient ischemic attack (TIA). The SAE in the Control participant was a fractured pelvis due to a fall on ice.

## 4 Discussion

To our knowledge, this is the first study to examine the combined effects of longer term exercise training with ibuprofen supplementation on muscle and bone in postmenopausal women using a factorial design. Resistance training promoted greater increases in hack squat and biceps curl strength and decreases in body fat than stretching. Contrary to our hypothesis, augmenting resistance training with ibuprofen did not have an additive effect on bone properties or muscle mass. Exercise training and/or ibuprofen supplementation had no effect on aBMD of the lumbar spine (L_1_–L_4_ vertebrae), femoral neck, or total body. There was a 6% decrease in aBMD of Ward's region in the control group compared to a 3% increase in the stretching and ibuprofen group, implying that ibuprofen helped maintain bone density at this site. Ward's region is clinically relevant as it is identified as the region at the femoral neck with the lowest aBMD (i.e. potentially the weakest). Collectively, these findings suggest that ibuprofen may have some beneficial bone effects for postmenopausal women.

Our results add to the limited body of research investigating the effects of ibuprofen supplementation and resistance training on aging muscle and bone biology. One notable study (Kohrt et al., 2010) demonstrated that 400 mg of ibuprofen given after resistance training sessions over nine months improved hip aBMD (total, trochanter, femoral neck and shaft) in *younger* women. However, recent research by this group demonstrated no significant effect on the aBMD in *older* adults regardless of whether supplementation occurred before or after resistance exercise training (Jankowski et al., 2015). The recent data presented by this group supports a trend for a deleterious effect of ibuprofen given after exercise (and before) in postmenopausal women, specifically at the hip (Jankowski et al., 2015). The authors recommended the cautious interpretations of their findings due to the lack of an exercise control group and drug dispensing error (Jankowski et al., 2015). Evident was the need for further studies in humans with greater sample size and follow-up time.

Our finding of a small beneficial effect of ibuprofen on bone (Ward's region) is supported by epidemiological studies that have found associations between regular NSAID use and improved bone density (Bauer et al., 1996, Carbone et al., 2003, Morton et al., 1998, Richards et al., 2006). Our study, however, showed no benefit when ibuprofen was consumed immediately after exercise sessions. An optimal intervention might be to alter the timing of the exercise bout and ibuprofen supplementation, perhaps taking ibuprofen at a time point during the day when one is not exercising (e.g. minimum eight hours before or after) or taking ibuprofen on non-exercise days.

Inhibition of prostaglandin synthesis by ibuprofen or other COX-inhibitors in animal studies has produced variable results on mechanically-loaded bone. The effect of ibuprofen may depend on the timing of administration with regards to the mechanical stimulus. Animal experiments suggest that ibuprofen supplementation before or during, but not after, loading impairs the bone response and adaptation to mechanical loading (including inflammation mediated bone formation) (Chow et al., 1998, Li et al., 2002). Thus, while prostanoids are theorized to be required for mechanically induced bone formation to occur, the prostanoid-dependent mechanism responsible for bone formation functions during mechanical loading, not after (Chow et al., 1998, Li et al., 2002). Collectively, animal studies suggest prostaglandin synthesis mechanically induced by COX-2 expression after loading does not contribute to bone formation. Ingesting ibuprofen after resistance training would therefore allow the prostanoid-dependent mechanism during loading to stimulate bone formation while blunting the increased production of harmful prostaglandins following exercise that may contribute to chronic inflammation. Our results, however, do not support either a detrimental or beneficial effect of ibuprofen supplementation after exercise loading of bone.

Ibuprofen and exercise were neither additive nor effective for increasing lean tissue mass. These findings are in support of prior studies showing no significant benefit to lean tissue mass when supplementing resistance training with 400 mg (versus placebo) of ibuprofen in younger or older women during resistance training programs (Kohrt et al., 2010, Candow et al., 2013, Krentz et al., 2008); however, potential exists for higher-ibuprofen dose to increase muscle mass in older adults who are participating in a resistance training program. It was demonstrated that 1200 mg of ibuprofen following resistance training increased lean tissue mass in older adults (Trappe et al., 2011). Perhaps the lack of improvement in lean tissue mass in our study and others (Kohrt et al., 2010, Candow et al., 2013, Krentz et al., 2008) is due to a lower dose (i.e. 400 mg versus 1200 mg). The low-dose of ibuprofen used, while safe and effective for increasing hip aBMD in premenopausal women (Kohrt et al., 2010), may not have been sufficient enough to counteract the resistance-training induced inflammatory response and catabolism which occurs in postmenopausal women. While the inflammatory and catabolic effects of exercise may lead to greater muscle protein synthesis and are thus beneficial in younger adults, this may not be the case with aging adults. While it is well accepted that resistance training prevents sarcopenia, evidence from animal studies suggest ibuprofen may be of benefit as well (Greig et al., 2009). A daily dosage of 30 mg kg^− 1^ prevented muscle wasting in old (i.e. 20 month old) rats; again this would translate to a much higher dose than used in our study and others if implemented in humans (e.g. 2220 mg for a 70 kg adult) (Rieu et al., 2009).

The effects of pro-inflammatory cytokines at the cellular and metabolic level should theoretically manifest as a measurable loss of muscle mass and strength and bone density and strength. There is dysregulation of inflammatory cytokines with age leading to over-production of pro-inflammatory cytokines such as interleukin-6 (IL-6) and tumor necrosis factor (TNF-α) (Degens, 2010). Precursors for these inflammatory cytokines include prostaglandins (i.e. PGE_1_ and PGE_2_) which are synthesized through the cyclooxygenase pathway from arachidonic acid mediated by the enzymes cyclooxygenase-1 and 2 (COX-1; COX-2) (Degens, 2010, Lopez-Otın et al., 2013). In our study, inflammation may have been reduced at the cellular and metabolic level and simply did not manifest into measurable changes at the tissue level. One could include measurements of the cellular and metabolic level over a shorter duration if a longer duration study is not feasible. However, an optimal intervention might require a longer duration, perhaps over 2 years, to allow changes to manifest at the tissue level.

The 4-group design of our study allowed the assessment of the interaction between ibuprofen and exercise training. An additional strength of our study was the high compliance in all intervention arms. However, our study had a number of limitations. Potential participants with high fracture risk were excluded via CAROC method, limiting generalizability. Ward's region aBMD was among five femoral neck measures assessed. We did not make adjustments for multiple comparisons; therefore, our Ward's region findings could be by chance (i.e. statistical error). Further, Ward's region is limited via overestimation of osteoporosis and should not be used in diagnosis (El Maghraoui and Roux, 2008). Our study was most likely underpowered to detect statistically significant differences between groups for primary variables. To detect ≥ 1% difference in aBMD of clinically relevant sites with 90% power each group would require approximately 40 per-protocol participants (2 sided, p ≤ 0.05); thus, to account for ~ 20% attrition, a sample size of 200 is required (Jankowski et al., 2015). Future research efforts should employ a similar study design with four recommended alterations: 1) increase to a daily dosage of 400 mg of ibuprofen, 2) alter the timing of the ibuprofen supplementation, so that supplementation does not occur within close proximity to the exercise, 3) lengthen the duration of the intervention, and 4) increase sample size to 200.

## 5 Conclusion

Ibuprofen supplementation immediately after resistance training did not have an additive effect on bone or muscle mass in postmenopausal women with low to moderate fracture risk. In contrast, our findings suggested that ibuprofen as administered by itself may have some benefit at Ward's region of the proximal femur. The timing and the dose of the ibuprofen may be important.

## Figures and Tables

**Table 1:** Baseline data by intervention group.

	ExIbu	Ex	Ibu	Control
(n = 23)	(n = 22)	(n = 23)	(n = 22)
Age (years)	65.4 (3.5)	65.3 (4.6)	65.5 (6.7)	65.0 (4.7)
Height (cm)	160.5 (4.7)	162.4 (5.7)	162.5 (6.6)	160.0 (6.6)
Lumbar spine aBMD (g/cm^2^)	0.989 (0.221)	0.906 (0.093)	0.941 (0.129)	0.978 (0.184)
Total hip aBMD (g/cm^2^)	0.860 (0.135)	0.836 (0.080)	0.865 (0.116)	0.844 (0.144)
Femoral neck aBMD (g/cm^2^)	0.703 (0.126)	0.681 (0.061)	0.722 (0.105)	0.697 (0.097)
Trochanter aBMD (g/cm^2^)	0.654 (0.097)	0.629 (0.062)	0.669 (0.100)	0.638 (0.105)
Intertrochanter aBMD (g/cm^2^)	1.034 (0.167)	0.996 (0.114)	1.026 (0.147)	1.021 (0.185)
Ward's aBMD (g/cm^2^)	0.542 (0.134)	0.510 (0.087)	0.541 (0.099)	0.549 (0.116)
Total body aBMD (g/cm^2^)	1.078 (0.090)	1.034 (0.091)	1.079 (0.104)	1.074 (0.131)
Narrow neck CSA (cm^2^)	2.75 (0.49)	2.67 (0.22)	2.92 (0.52)	2.74 (0.35)
Narrow neck SPW (cm)	3.41 (0.33)	3.34 (0.28)	3.41 (0.30)	3.39 (0.34)
Narrow neck Z (cm^3^)	1.35 (0.35)	1.27 (0.18)	1.46 (0.35)	1.33 (0.19)
Intertrochanter CSA (cm^2^)	4.92 (0.90)	4.78 (0.59)	5.02 (0.81)	4.61 (1.04)
Intertrochanter SPW (cm)	5.53 (0.40)	5.76 (0.49)	5.81 (0.35)	5.53 (0.78)
Intertrochanter Z (cm^3^)	4.30 (0.93)	4.32 (0.74)	4.64 (0.88)	4.15 (1.03)
Femoral shaft CSA (cm^2^)	4.15 (0.65)	4.16 (0.44)	4.23 (0.67)	3.94 (0.56)
Femoral shaft SPW (cm)	3.01 (0.23)	3.01 (0.23)	3.02 (0.22)	3.01 (0.20)
Femoral shaft Z (cm^3^)	2.39 (0.41)	2.39 (0.31)	2.49 (0.48)	2.28 (0.47)
Total Mass (kg)	73.95 (12.91)	71.02 (11.66)	76.08 (13.73)	75.49 (14.98)
Lean mass (kg)	40.33 (5.33)	40.86 (5.13)	42.64 (5.98)	40.88 (5.11)
Fat mass (kg)	30.33 (8.14)	28.15 (7.96)	31.39 (8.88)	32.51 (10.31)
Body fat percentage (%)	42.03 (4.66)	39.07 (5.49)	40.59 (5.71)	42.15 (5.94)
Average tandem walk time (s)	52.39 (15.41)	56.00 (20.75)	52.59 (18.21)	55.49 (23.43)
Average tandem walk errors	5.11 (5.56)	3.10 (3.89)	5.29 (6.62)	3.81 (3.72)
Biceps curl 1RM (kg)	9 (1)	8 (1)	9 (2)	8 (2)
Hack squat 1RM (kg)	38 (25)	45 (30)	54 (34)	41 (20)
LTEQ score	20 (11)	20 (13)	28 (25)	21 (19)
Total energy intake	1775 (740)	1614 (485)	1726 (568)	1581 (377)
Calcium intake (mg/d)	869 (305)	787 (343)	922 (468)	729 (236)
Vitamin D intake (IU)	129(73)	142 (128)	193 (159)	155 (134)

All values are means (SD); SD = standard deviation.

Abbreviations: BMD = bone mineral density; CSA = cross-sectional area; SPW = subperiosteal width; Z = section modulus; 1RM = 1-repitition maximum; LTEQ = leisure time exercise questionnaire.

**Table 2:** Mean absolute changes (95% CI) from baseline to 9 months for areal bone mineral density within groups.

	ExIbu (n = 18)		Ex (n = 19)		Ibu (n = 17)		Control (n = 15)		Exercise	Supplement	Interaction
Change	95% CI	Change	95% CI	Change	95% CI	Change	95% CI	p-Value	p-Value	p-Value
Lumbar spine	− 0.007	(− 0.024, 0.009)	− 0.003	(− 0.016, 0.010)	0.005	(− 0.009, 0.019)	0.011	(− 0.007, 0.029)	0.069	0.482	0.883
Total hip	0.003	(− 0.011, 0.018)	0.008	(− 0.006, 0.022)	0.007	(− 0.007, 0.021)	0.001	(− 0.012, 0.013)	0.778	0.912	0.415
Femoral neck	− 0.010	(− 0.033, 0.014)	− 0.002	(− 0.017, 0.014)	− 0.006	(− 0.022, 0.009)	− 0.023	(− 0.037, − 0.010)	0.285	0.600	0.148
Trochanter	0.001	(− 0.009, 0.011)	0.004	(− 0.007, 0.015)	0.006	(− 0.008, 0.020)	0.002	(− 0.006, 0.011)	0.759	0.983	0.504
Intertrochanteric	0.007	(− 0.016, 0.030)	0.010	(− 0.012, 0.033)	0.005	(− 0.009, 0.018)	− 0.006	(− 0.031, 0.019)	0.369	0.719	0.511
Ward's region	− 0.022	(− 0.056, 0.012)	− 0.003	(− 0.034, 0.029)	0.017	(− 0.006, 0.040)^a^	− 0.035	(− 0.063, − 0.006)^a^	0.786	0.261	0.015
Total body	0.005	(− 0.005, 0.015)	0.002	(− 0.008, 0.012)	− 0.003	(− 0.013, 0.008)	− 0.007	(− 0.015, 0.001)	0.092	0.438	0.889

All values are mean absolute changes (95% CI) in g/cm2; CI = confidence interval.

Exercise, ibuprofen, and interaction effects are presented in last two columns.

a Ibu different from Control groups (post hoc; p = 0.017).

**Table 3:** Mean absolute changes (95% CI) from baseline to 9 months for hip structural analysis within groups.

	ExIbu (n = 18)		Ex (n = 19)		Ibu (n = 17)		Control (n = 15)		Exercise	Supplement	Interaction
Change	95% CI	Change	95% CI	Change	95% CI	Change	95% CI	p-Value	p-Value	p-Value
Narrow neck CSA (cm^2^)	− 0.03	(− 0.15, 0.08)	− 0.04	(− 0.13, 0.05)	− 0.09	(− 0.20, 0.02)	− 0.06	(− 0.12, 0.01)	0.432	0.790	0.708
Narrow neck SPW (cm)	− 0.04	(− 0.13, 0.05)	0.10	(0.01, 0.19)	0.02	(− 0.13, 0.16)	0.05	(− 0.04, 0.13)	0.977	0.076	0.250
Narrow neck Z (cm^3^)	0.00	(− 0.08, 0.09)	− 0.02	(− 0.11, 0.07)	− 0.03	(− 0.13, 0.06)	− 0.02	(− 0.08, 0.05)	0.708	0.911	0.630
Intertrochanteric CSA (cm^2^)	0.05	(− 0.08, 0.18)	0.08	(− 0.13, 0.28)	− 0.06	(− 0.23, 0.12)	0.23	(− 0.31, 0.77)	0.868	0.257	0.331
Intertrochanteric SPW (cm)	0.02	(− 0.09, 0.14)	0.11	(− 0.02, 0.24)	− 0.02	(− 0.22, 0.18)	0.22	(− 0.21, 0.65)	0.770	0.146	0.490
Intertrochanteric Z (cm^3^)	0.10	(− 0.08, 0.29)	0.15	(− 0.15, 0.45)	− 0.01	(− 0.25, 0.23)	0.20	(− 0.24, 0.64)	0.842	0.370	0.569
Shaft CSA (cm^2^)	0.06	(− 0.02, 0.14)	− 0.01	(− 0.09, 0.07)	− 0.03	(− 0.12, 0.07)	0.17	(− 0.20, 0.53)	0.609	0.439	0.118
Shaft SPW (cm)	− 0.02	(− 0.07, 0.03)	− 0.03	(− 0.07, 0.01)	− 0.03	(− 0.08, 0.02)	0.01	(− 0.07, 0.10)	0.526	0.548	0.339
Shaft Z (cm^3^)	0.02	(− 0.03, 0.07)	− 0.02	(− 0.08, 0.04)	− 0.06	(− 0.14, 0.02)	0.14	(− 0.17, 0.46)	0.572	0.258	0.083

All values are mean absolute changes (95% CI); CI = confidence interval.

Exercise, ibuprofen, and interaction effects are presented in last two columns.

Abbreviations: CSA = cross-sectional area; SPW = subperiosteal width; Z = section modulus; Intertroch = intertrochanter.

**Table 4:** Mean absolute changes (95% CI) from baseline to 9 months for body composition, balance, and strength.

	ExIbu (n = 18)		Ex (n = 19)		Ibu (n = 17)		Control (n = 15)		Exercise	Supplement	Interaction
Change	95% CI	Change	95% CI	Change	95% CI	Change	95% CI	p-Value^a^	p-Value	p-Value
Fat mass (kg)	− 1.07	(− 2.01, − 0.12)	− 0.03	(− 1.08, 1.01)	0.45	(− 0.75, 1.65)	0.56	(− 0.23, 1.35)	0.033	0.241	0.345
Lean tissue mass (kg)	0.00	(0.00, 0.00)	0.97	(0.33, 1.66)	0.20	(− 0.38, 0.78)	0.44	(− 0.48, 1.36)	0.237	0.248	0.660
Body fat percentage (%)	− 1.21	(− 2.26, − 0.16)	− 0.54	(− 1.35, 0.28)	0.20	(− 0.81, 1.21)	0.12	(− 0.53, 0.77)	0.019	0.493	0.385

*Strength*											
Biceps curl (kg)	2	(1, 2)	2	(1, 3)	0	(0, 1)	1	(0.1)	< 0.001	0.248	0.455
Hack squat (kg)	49	(31, 67)	40	(25, 55)	7	(0, 14)	6	(− 1, 12)	< 0.001	0.966	0.830

*Dynamic balance*											
Average time (s)	− 5.21	(− 11.71, 1.29)	− 12.47	(− 22.39,-2.54)	− 7.86	(− 13.12, − 2.61)	− 4.57	(− 14.83, 5.70)	0.509	0.618	0.186
Average errors	0.25	(− 1.23, 1.73)	0.17	(− 1.02, 1.35)	− 0.53	(− 1.72, 0.66)	− 0.12	(− 1.37, 1.14)	0.390	0.787	0.685

All values are mean absolute changes (95% CI); CI = confidence interval.

Exercise, ibuprofen, and interaction effects are presented in last two columns.

Dynamic balance is average of two attempts.

a p-value for the main effect; Resistance training improved compared to stretching groups.

**Table 5:** Mean absolute changes (95% CI) from baseline to 9 months for descriptive outcome variables.

	ExIbu (n = 18)		Ex (n = 19)		Ibu (n = 17)		Control (n = 15)		Exercise	Supplement	Interaction
Change	95% CI	Change	95% CI	Change	95% CI	Change	95% CI	p-Value^b^	p-Value	p-Value
Total energy intake (kcal/d)	− 119	(− 315, 77)	72	(− 133, 276)	− 118	(− 502, 267)	− 188	(− 394, 18)	0.047	0.165	0.596
Total protein intake (g/d)	− 5	(− 14, 4)	− 1	(− 10, 8)	− 5	(− 19, 9)	− 7	(− 16, 3)	0.209	0.329	0.862
Total fat intake (g/d)	− 2	(− 16, 12)	8	(− 5, 20)	− 5	(− 22, 13)	− 8	(− 19, 2)	0.039	0.253	0.654
Total carbohydrate intake (g/d)	− 22	(− 40, − 4)	− 1	(− 23, 21)	− 12	(− 52, 28)	− 25	(− 52, 1)	0.143	0.289	0.377
Calcium intake (mg/d)^a^	− 83	(− 186, 19)	− 27	(− 146, 97)	− 107	(− 349, 134)	− 145	(− 226, − 63)	0.073	0.391	0.959
Vitamin D (μg/d)^a^	14	(− 33, 60)	− 47	(− 88, − 7)	− 41	(− 120, 37)	− 4	(− 63, 55)	0.583	0.949	0.024
Leisure physical activity score (arbitrary units)	− 1	(− 12, 9)	1	(− 6, 7)	2	(− 7, 12)	9	(− 6, 25)	0.211	0.362	0.591

All values are mean absolute changes (95% CI); CI = confidence interval.

Exercise, ibuprofen, and interaction effects are presented in last two columns.

a Values only include nutrients from dietary intake and do not include the supplements given during the study.

b p-value for the main effect; Stretching group decreased total energy and fat intake compared to resistance training group.
